# Health-related on-pack communication and nutritional value of ready-to-eat breakfast cereals evaluated against five nutrient profiling schemes

**DOI:** 10.1186/1471-2458-14-1178

**Published:** 2014-11-19

**Authors:** Gesa Maschkowski, Monika Hartmann, Julia Hoffmann

**Affiliations:** Institute for Food and Resource Economics, Bonn University, Nussallee 21, Building 2, Bonn, 53115 Germany; Import Promotion Desk, Am Weidendamm 1A, Berlin, 10117 Germany

**Keywords:** Nutrient profiles, Breakfast cereals, Health claims, Consumer information, Food labelling, Food legislation

## Abstract

**Background:**

There is an ongoing debate regarding health-related on-pack information appearing on products with low nutritional quality. The purpose of the study was to contribute to this discussion by examining the relationship between health-related on-pack information and the overall nutritional value of highly processed ready-to-eat breakfast cereals (RTECs).

**Methods:**

Maximum variation sampling was used to select 128 highly processed RTECs in Germany in 2010. In 2012, two additional samples were collected in Norway (*n* =38) and Germany (*n* =73) to allow for comparisons of products from countries with different regulations concerning nutrient profiles. All products were evaluated against five nutrient profiling models from government-related agencies. Mann–Whitney U Tests and Chi-square statistics was used to compare the nutrient profiles of different product categories. Logistic regression analysis was conducted to identify on-pack information on German products predicting a satisfactory nutritional profile.

**Results:**

The majority of RTECs displayed health-related information on the packaging, but only 4–36% of German products met the criteria of the different nutrient profiles. The rate was lower for cereals marketed to children, Norwegian cereals performed better (36-64%). Health-related on-pack information was not consistently related to the nutrient profiles. The following on-pack criteria predicted a satisfactory nutrient profile on RTECs of the German 2010 sample: i) cereals not marketed to children, ii) clean labelling (free-from claims) and possibly organic labelling and whole grain claims.

**Conclusions:**

Our results suggest that the implementation of a mandatory nutrient profiling scheme for products with health-related on-pack information could contribute to a consistent relationship between health-related information on RTECs and the overall nutritional value of the product. Improvements should also consider the provision of a simple nutritional labelling scheme on the front of the packaging, standardized serving sizes, accurate product names, and clearly defined whole grain claims.

## Background

Which product contains more dietary fibre, crispy roasted cornflakes with vitamins or crispy rice and whole grain wheat flakes with chocolate, vitamins, calcium, and fortified with iron? Surprisingly, the cornflakes contain more fibre (3.7 g per 100 g) than the rice and whole grain flakes (3.0 g per 100 g) because of the low whole grain content of the latter. This example of two products purchased in Germany in 2010 illustrates how product names containing health-related information can cause consumers’ confusion at the point of sale. As packaging is commonly used by consumers to judge product quality and to support their decision-making process at the point of sale [1], promotional marketing on food packaging has been shown to influence children’s and adults’ preferences and purchase decisions [2]. For example, Roberto et al. found that children preferred the taste of products in packaging displaying licensed characters [3], and McNeal and Ji showed that children were aware of brand names and could draw a close likeness of a particular brand of cereal packaging [4].

Previous research indicates that consumers expect products with nutrition and health claims on the packaging to have a better overall nutritional value compared with products without such information: Harris and colleagues reported that parents considered children’s cereals with health-related claims to be more nutritious and to provide specific health-related benefits for their children, resulting in a greater willingness to buy these cereals [5]. Children are similarly influenced as Soldavini et al. showed that fourth- and fifth-grade children perceive products from packaging with nutrition claims as being healthier than those without such claims [6].

The European Regulation No. 1924/2006 of the European parliament and council, the so-called Regulation on Health and Nutrition Claims, aims to avoid a situation where nutrition or health claims may mask the overall nutritional status of a food product and mislead consumers when trying to make healthy choices. This regulation proposes application of a nutrient profile for assessing the eligibility for health and nutrition claims [7]. Nutrient profiling of foods can be defined as the “the science of categorising foods according to their nutritional composition” [8]. Nutrient profiling systems have been proposed for various purposes, such as regulating commercial food marketing to consumers, identifying the eligibility of food products to show nutrition or health claims, and promoting reformulation of food products or food labelling [9]. However, 8 years after the implementation of Regulation 1924/2006, hundreds of nutrition and health claims have been authorised, or are in the process of authorisation, and no nutrient profile is in place yet. Therefore, in most European countries, health-related information appears on the packaging independent of the overall nutritional value of the product. An exception is the Keyhole, which is a government-approved nutrient profiling model applied in Denmark, Sweden, and Norway [10].

Cereals in general and breakfast cereals in particular, are marketed using a wide range of nutrition and health-related information on the packaging, such as nutrition tables, nutrition and health claims or dietary recommendations [11–14]. To the best of our knowledge, there is little published evidence available on the diversity of health related on-pack information and the relationship between this information and the overall nutritional value of breakfast cereals measured by different nutrient profiling schemes. The purpose of the study was to address this research gap by exploring the current health-related labelling practice on highly processed ready-to-eat cereals (RTECs) and assessing the healthiness of the products. In particular, the study aimed to answer the following questions: i) what kind of health-related information is depicted on the packaging of RTECs; ii) what is the overall nutritional value of RTECs and do differences exist in the nutrient profiles of products that vary according to the target customers (children, adults), the type of health and nutritional information displayed on the packaging (claim versus no claim) and countries with different regulations concerning nutrient profiles (Norway and Germany); and iii) is there on-pack information that can be used by consumers as an external cue for predicting a satisfactory nutrient profile? The present study will provide evidence on the potential impact of implementation of a nutrient profiling scheme in the EU as proposed by the European Regulation 1924/2006.

## Methods

### Sample one: RTECs in Germany in 2010

In our study we focused on highly processed ready-to-eat cereals (RTECs) such as flakes, puffed and extruded cereals. Oat flakes and any kind of muesli were excluded from the study as Hampshire et al. [15] demonstrated that the fibre and sugar content of muesli products significantly differ from highly processed RTECs. Our pre-study also showed that muesli on-pack information varies from the information on highly processed cereals. Thus, RTECs constitute a separate product category, characterized by a large range of products from various manufacturers offered through different commercial chains.

The heterogeneity of manufacturers and retailers in Germany and regional differences made sampling the entire RTEC food category infeasible. Maximum variation sampling was used to select RTECs, representing a wide range of variation in our dimensions of interest – different manufacturers, producers or retailers, production methods, heterogeneous nutrient contents, and different customer target groups (adults or children). To cover a broad spectrum of products, we first, included different retail formats in our analysis (three discounters, one department store with more than 5,000 m^2^, one regular supermarket with less than 5,000 m^2^) and two whole food grocers. The stores were located in the city of Bonn or its surroundings. Second, companies from the German Cereal and Oat Milling Association were asked to provide product or package samples of every RTEC they offer. From these two sampling methods we collected products from 21 different manufacturers. The majority of brands offered products with one to three different flavours, however, some offered more than three different taste variants. We recorded all available cereals, but we limited the sample to three different flavours from one product in our analysis because more variants of highly similar products did not contribute to our main research question. Sample one consisted of 128 RTECs in total, with 58 products (45%) classified as marketed to children in accordance with criteria developed by Düren and Kersting [16].

### Sample two and three: RTECs in Germany and Norway in 2012

To investigate differences in the overall nutritional value in RTECs between a country with a government-approved nutrient profile and a country without, we drew a second sample in Germany in 2012 and a third one in Norway in 2012. They were collected from two discount stores and two supermarkets (less than 5000 m^2^ because of their inner-city location) in cities of Bonn and Trondheim. In total, 73 RTECs were selected in Germany and 38 RTECs in Norway. The Norwegian sample was smaller, because of a narrower range of products in Norwegian supermarkets.

### Selected nutrition profiles used in nutritional evaluation

We focused on five nutrient profiling systems that were developed by government agencies. We selected nutrient profiling models that were applied for different purposes and target groups to enhance the reliability of the results. The model of the Interagency Working Group (IWG) [17] and the UK model (OFCOM) [18] were developed to regulate food marketing to children. These models contribute to evaluation of RTECs for children. They are based on different evaluation systems: The IWG applies a threshold model, whereas the UK model is based on a scoring system. The EU system [19] and the Food Standards Australia and New Zealand model (FSANZ) [20] aim to identify the eligibility of food products to show nutrition claims and/or health claims. They are also based on different calculation systems. Finally, the Nordic Keyhole [10] is applied for food labelling in Scandinavia. The Nordic Keyhole imposes the strictest requirements for nutrient content of cereals, whereas the EU model is the least demanding model (see Table 1 for details). The OFCOM model and the FSANZ model rate foods on a scale from −15 (most healthy) to +40 (least healthy). The nutrient profiling scheme established by FSANZ is a less demanding version of the original UK model, allowing a higher score for “baseline points” from energy, saturated fat, total sugar, and sodium content of food.

**Table 1:** **Main characteristics of five governmental led nutrient profiling systems**

Name of the system	Field of application	Nutrients to limit per 100 g*	Ingredients and nutrients to encourage per 100 g*	Calculation
Keyhole	Labelling (in use)	Total sugars (13 g), refined sugars (10 g), fat (7 g), sodium (0.5 g).	Fibre (6 g), 50% whole grain at minimum.	Threshold, category-specific
IWG	Advertising to children eligibility (draft)	Added sugars (26.7 g), saturated fat (3.3 g) and 15% less of calories for individual food, trans fat (0 g), sodium (0.7 g).	50% whole-grain, fruit, vegetables and nuts at minimum.	Threshold, category-specific
EU-System	Claim eligibility (draft)	Sugars (25 g), saturated fat (5 g), sodium (0.5 g).	None	Threshold, category-specific
OFCOM	Advertising to children eligibility (in use)	Energy, saturated fat, total sugar, sodium.	Fruits, vegetables and nuts, fibre, protein.	Scoring, across the board
FSANZ	Health claim eligibility (in use)	Energy, saturated fat, total sugar, sodium.	Fruits, vegetables and nuts, fibre, protein.	Scoring, across the board

*Abbreviations*: *OFCOM* Office of Communication, *FSANZ* Food Standards Australia and New Zealand, *EU* European Union, *IWG* Interagency Working Group.

*In the case of category-specific threshold the ones relevant for cereals were used.

### Coding

In 2010, we started a pre-study with the support of graduate students, outlining a preliminary coding form and conducting the first descriptive analyses of RTEC packaging. In 2011, the authors extended, tested and refined the research questions and coding forms. From this, the 2010 sample was coded for 23 main topics and several subtopics, such as target group, words used within the product name, location of the product name, public or private labels, nutrition and health claims, nutrition labelling, GDA (guideline daily amounts), serving size and nutrients. For definitions of predictive variables see Table 2. The 2012 samples were coded for energy, nutrients, whole grain content and health-related on-pack information.

**Table 2:** **Types and definitions of health-related information on RTECs from three samples (Germany 2010, Germany 2012, Norway 2012)**

Variable	Definition	German 2010 sample	German 2012 sample	Norwegian 2012 sample
		***n*** = 128	***n*** = 73	***n*** = 38
		% (n)	% (n)	% (n)
Nutrition claim	Nutrition claims, defined by Regulation (EC) No. 1924/2006, state, suggest or imply that a food has particular beneficial nutritional properties because of energy, nutrients or other substances [7].	58 (74)	22 (16)	50 (19)
Health claim	Health claims, defined by Regulation (EC) No. 1924/2006, state, suggest or imply that there is a relationship between a food category or one of its constituents and health [7] such as positive effects of the RTEC on digestion or weight management.	7 (9)	0	11 (4)
Whole grain claim	Whole grain claims, not yet regulated by EU-Regulation 1924/2006, include whole grain related on-pack information, such as “xy% whole-grain”, “increased share of whole grain”, “whole wheat”, “whole oat” or whole grain labels. Cereals with such claims have a high variation in their share of whole grain content.	31 (39)	37 (27)	45 (17)
Clean labelling	There is no legal definition for clean labelling per se. It is generally understood to mean eye-catching claims indicating the product is free from negative-sounding ingredients, such as food additives, allergens, genetically modified organisms or nutrients such as sugar or salt [35].	12 (15)	8 (6)	11 (4)
Healthy ingredients in product name	According to the Regulation (EU) No. 1169/2011, a product name is required for every product marketed in Europe [36]. The product name of breakfast cereals usually consists of several words describing the qualities of the product. The item “healthy ingredients in product name” was coded when the product name included healthy ingredients such as vitamins, minerals, dietary fibre or whole grain.	57 (73)	56 (41)	37 (14)
Organically certified	Words and labels indicating “organic”, “bio” or “eco” are defined by regulation (EC) No. 834/2007 on organic production and labelling of organic products [37].	9 (11)	1 (1)^a^	0^a^
GDA	GDA, guideline daily amounts, is a nutrition fact label indicating the contribution of one portion (serving) of food in terms of energy (calories), sugar, fat, saturated fats and sodium to a person's daily intake guideline. The majority of the GDA labels are displayed on the front of the package.	76 (97)	85 (62)	53 (20)

^a)^The 2012 sample did not include organic shops.

Two students, who had completed approximately 20 hours of formal training over a two-month period, conducted the packaging coding. Intercoder reliability was assessed by Holsti’s method [21], with scores ranging from .80 to .98. The overall agreement between coders was .92. These scores are good and in accordance with publication standards for refereed journals [22]. Moreover, coding of health and nutrition claims was carefully reviewed by one of the authors (GM). All nutrient data were obtained directly from the cereal packaging, from manufacturers’ websites or provided directly by the manufacturers. Our aim was not to validate or complement these data by our own analysis, but to use the same information available to consumers when trying to identify the nutritional value of the product. The data were complete for the German samples except for trans fatty acids, which are required by the nutrient profiling scheme of the IWG (Table 1). Trans fatty acids are not subjected to standard food analysis or labelling in Germany; however, recent analysis found that breakfast cereals in Western Europe had low values of trans fatty acids compared with other food products [23, 24]. We therefore concluded that the IWG model is still informative for the purposes of our study. Moreover, the packaging rarely indicated the amount of added sugars as required by the IWG model. Therefore, the amount of total sugar depicted on the packaging was used. For calculation of the Keyhole score, we considered the threshold on total sugar, but we did not include the threshold for refined sugar because the latter was not available on cereal packages. One Norwegian product (puffed sweetened wheat) from the 2012 samples did not display its main nutrients and was excluded from the calculations of OFCOM score and the FSANZ score. Based on the nutrient content of a comparable Norwegian product it scored “0” in the remaining profiling schemes.

### Statistical analysis

We first assessed the nutrient content (energy, nutrients and ingredients) and OFCOM scores of RTECs marketed to children compared with others for the German sample in 2010 as well as for products purchased in Norway and Germany in 2012. Mann–Whitney U Tests were used to compare the nutrient content and OFCOM scores of these groups.

Our next step was to evaluate the nutritional value of RTECs based on the five nutrient profiling systems. We differentiated between several RTEC categories, such as RTECs for children, for adults, RTECs with different health and nutrition claims, RTECs with and without organic certification, as well as RTECs purchased in Norway and Germany. Chi-square statistics were applied to assess the relationship between RTEC categories and their nutrient profiles.

In addition, the prices of the 2010 sample were analysed to determine whether they signal the overall nutritional value of a product. Spearman’s rank correlation coefficient was used to measure the strength of the associations between the product price and the OFCOM score as well as the FSANZ score. Then, we created a dichotomous variable for products with prices above the median (Highprice ≥50 Euro cent/100 g =1). Otherwise, the products were coded as “0” on this variable (Highprice <50 Euro cent/100 g =0). We used Chi-square test to identify the share of products with satisfactory nutrient profiling scores and low price.

The last step was to explore whether packaging information allows consumers in Germany to detect healthy RTECs. Two multivariate logistic regression models were applied to identify on-pack information that predicts a recommended nutrient profile. Two dependent variables measured the nutritional value of RTECs. The first variable predicted a satisfactory OFCOM score as a function of predictor variables given on the packaging: an OFCOM score greater than four suggested products met the criteria of OFCOM, whereas an OFCOM score less than four was applied for products classified as less healthy. The second variable was created to predict the event that a product fits at least one nutrient profile or did not fit any of the five nutrient profiles. Based on our previous analysis, we selected on-pack criteria which seemed to be connected to the nutritional value of RTECs as independent variables: marketing to children, healthy ingredients in product name, whole grain claims, health and/or nutrition claims, clean labelling and organically certified (see definitions in Table 2). If a *p*-value was found to be less than 5% (*p* <0.05) then the result was considered statistically significant. An observed *p*-value less than 1% (*p* <0.01) was interpreted as highly statistically significant.

The data were analysed using Statistical Package for the Social Sciences (SPSS; Version 21.00) as well as STATA (Version 12.0).

## Results

### Health-related information on cereal packaging

Many products in the German 2010 sample (*n* = 128) were marketed as healthy products, with 84% (*n* = 107) displaying any kind of health-related information on the packaging such as nutrition claims, health claims, whole grain claims, clean labelling or healthy ingredients in the product name. Some products carried several claims. In particular, 58% (*n* = 74) of RTEC packaging contained nutrition claims, 7% (*n* = 9) displayed health claims and 12% (*n* = 15) applied a clean labelling claim (see Table 2). Whole grain claims were made by 31% (*n* = 39) of RTECs, which contained between 7–93% whole grain. The whole grain content of the entire German 2010 sample ranged between 7–100%. Furthermore, 57% (*n* = 73) of products collected in Germany made reference to healthy ingredients in the product name, such as vitamins and minerals and/or whole grain. The packaging of the German 2012 sample (*n* = 73) displayed less nutrition claims (22%, *n* = 16), no health claims but slightly more whole grain claims (37%, *n* = 27), which contained between 14–95% whole-grain. Content analysis of the Norwegian 2012 sample (*n* = 38) generated slightly different result. Half of the products displayed nutrition claims (50%, *n* = 19), a share of 11% (*n* = 4) made use of health claims, 29% (*n* = 11) depicted the Nordic Keyhole and 11% (*n* = 4) of the Norwegian RTECs packaging applied a “free from claim”. Whole grain claims were displayed on 45% (*n* = 17) of RTECs. They contained between 39-100% whole grain (see Table 2). A share of 26% (*n* = 10) products displayed both wholegrain claim and Nordic Keyhole.

### Serving sizes

The majority of cereal packages, particularly those from Germany, displayed the guideline daily amount (GDA) signalling system (see Table 2): We found the GDA system on 76% (*n* = 97) of the German 2010 sample, on 85% (*n* = 62) of the German 2012 sample and on 53% (*n* = 20) of packaging purchased in Norway 2012. GDA information on children’s products referred to the daily intake guidelines of adults. The mean volume of the recommended serving size of children’s cereals purchased in 2010 was slightly lower (30.2 g) than the mean volume of non-children’s cereals (35.8 g). Serving sizes varied between 30 g to 60 g for the German 2010 sample, between 30 g to 100 g for the German 2012 sample and between 30 g to 45 g for the Norwegian 2012 sample.

### Nutritional content of RTECs

RTECs advertised to children from the German 2010 sample (*n* = 58) had significant higher OFCOM scores compared with non-children RTECs (*n* = 70, *p* = 0.000) (see Table 3). They were significantly higher in sugar (*p* = 0.000) and lower in fibre (*p* = 0.005) as compared with other RTECs. There was also a significant difference between the nutrition profiles of the German and Norwegian RTECs sampled in 2012. Cereals purchased in Norway were on average lower in energy (*p* = 0.000), lower in sugar (*p* = 0.008), higher in dietary fibre (*p* = 0.019) and had a much lower OFCOM score (*p* = 0.000).

**Table 3:** **Nutrients and OFCOM score of different RTEC categories and samples**

Parameter		Energy (kcal/100 g)	Sugar (g/100 g)	Fat (g/100 g)	Satfat (g/100 g)	Fibre (g/100 g)	Sodium (g/100 g)	OFCOM Score
Children’s RTECs 2010	mean	391.6	32.3	5.1	2.16	3.9	0.30	11.7
(*n* = 58)	SD^a^	23.4	7.8	4.6	2.30	1.6	0.19	3.8
Non-children’s RTECs 2010	mean	377.9	22.0	4.5	1.60	6.1	0.35	7.8
(*n* = 70)	SD	30.5	11.9	4.3	1.83	4.0	0.26	6.3
Mann–Whitney-*U* test	p^b^	0.001	0.000	0.395	0.068	0.005	0.274	0.000
Norwegian 2012 (*n* = 38)	mean	374.9	18.1	3.4	0.80	7.6	0.30	4.5
	SD	17.4	13.0	2.3	0.55	5.4	0.19	5.0
German 2012 (*n* = 73)	mean	389.8	24.9	4.6	1.76	5.1	0.34	9.2
	SD	24.8	10.8	4.3	1.86	2.8	0.25	5.4
Mann–Whitney-*U* test	p^b^	0.004	0.008	0.660	0.119	0.010	0.855	0.000

^a)^SD = Standard Deviation, ^b)^p = Significance Level.

### Evaluation of the nutritional value of RTECs against five profiling models

Few of the products from the German 2010 sample met the criteria of the different nutrient profiles (4-28%, *n* = 5-29). The results (see Table 4) were differentiated by grouping the cereals into different categories:

**Table 4:** **Percentage of RTECs that met the respective nutrient profiles by sample, target group and claim type**

Parameter	Keyhole	IWG	EU	OFCOM	FSANZ
	yes	yes	yes	yes	yes
	% (n)	% (n)	% (n)	% (n)	% (n)
All German RTECs 2010 (*n* = 128)	4 (5)	16 (20)	28 (36)	14 (18)	23 (29)
***Products by target (sample 2010)***					
Children RTECs (*n* = 58)	0 (0)	0 (0)	9 (5)	2 (1)	9 (5)
Non-Children RTECs (*n* = 70)	7 (5)	30 (21)	44 (31)	24 (17)	34 (24)
Chi-square test	a	0.000	0.000	0.000	0.001
***Products by claim type (sample 2010)***					
Nutrition or Health Claim (*n* = 77)	5 (4)	22 (17)	35 (27)	16 (12)	26 (20)
No Nutrition or Health Claim (*n* = 51)	2 (1)	8 (4)	18 (9)	12 (6)	18 (9)
Chi-square test	a	0.049	0.044	0.612	0.291
Whole grain claim (*n* = 39)	10 (4)	41 (16)	46 (18)	21 (8)	36 (14)
No whole grain claim (*n* = 89)	1 (1)	6 (5)	20 (18)	11 (10)	17 (15)
Chi-square test	a	0.000	0.005	0.177	0.023
Clean labelling (*n* = 15)	13 (2)	27 (4)	40 (6)	40 (6)	47 (7)
No clean labelling (*n* = 113)	3 (3)	15 (17)	27 (30)	11 (12)	19 (22)
Chi-square Test	a	a	a	a	a
***Healthy ingredients mentioned in product names (sample 2010)***					
Any healthy ingredient (*n* = 73)	6 (4)	23 (17)	33 (24)	15 (11)	25 (18)
No healthy ingredient (*n* = 55)	2 (1)	7 (4)	22 (12)	13 (7)	20 (11)
Chi-square Test	a	0.017	0.233	0.801	0.670
***Products by production method (sample 2010)***					
Organically certified (*n* = 11)	9 (1)	18 (2)	46 (5)	46 (5)	55 (6)
Conventional (*n =* 117)	3 (4)	16 (19)	27 (31)	11 (13)	20 (23)
Chi-square Test	a	a	a	a	a
***Products by country (sample 2012)***					
German RTECs 2012 (*n* = 73)	7 (5)	16 (12)	36 (26)	14 (10)	26 (19)
Norwegian RTECs 2012 (*n* = 38)	32 (12)	40 (15)	66 (25)	41 (15)^b^	60 (22)^b^
Chi-square Test	0.001	0.010	0.003	0.003	0.001

^a)^Chi-square Test could not be applied, expected cell frequencies were below the adequate expected counts of 5 ^b)^n = 37.

The proportion of German children’s cereals (*n* = 58) that fit the two nutrient profiles developed to regulate food marketing to children was small: Only 2% (*n* = 1) could be marketed to children according to OFCOM criteria. None of them met the criteria of the IWG model because of their low fibre content. However, 33% (4 products out of 12) of the Norwegian children’s cereals met the OFCOM criteria and 23% (3 products out of 13) met the criteria of IWG. A total of 15% (2 products out of 13) of the Norwegian children’s cereals met the criteria of the Keyhole model (results not shown in Table 4).

The majority of RTECs from German 2010 sample displaying health and/or nutrition claims (*n* = 77) did not meet the requirements of the selected profiling schemes. Depending on the profiling scheme, the proportion meeting the requirements was between 5% (*n =* 4) and 35% (*n* = 27). These proportions increased when products with claims of whole grain or clean labelling were evaluated. RTECs with nutrition and health claims did not score better according to the FSANZ model compared with RTECs without nutrition and health claims. However, according to the criteria of the EU model this difference was significant.

RTECs of the German 2010 sample mentioning any healthy ingredients in the product name did qualify more often against the IWG but not against the EU, the FSANZ and the OFCOM models than those without any reference to healthy ingredients.

Organically certified RTECs more often met the criteria of the OFCOM and the FSANZ models compared with conventional products.

Comparison of the Norwegian and German 2012 samples showed that the proportion of products that met the nutrient profiles was significantly higher for the Norwegian sample than for the German sample. This applied for all nutrient profiles that were considered in the analysis.

### Price and nutritional value of German 2010 sample (n = 128)

Spearman’s rank correlation showed a moderate but highly significant negative association between price and OFCOM score (*R* = −0.294, *p* =0.001) and price and FSANZ score (*R* =-0.288, *p* = 0.001), suggesting that a higher nutrient profiling score is linked to a higher price. These results were confirmed by Chi square test demonstrating for example that only two products out of 18 that met the OFCOM criteria belonged to the low price category (expected cell frequency for this category was 9, *p* = 0.001). Both products were store brands, not marketed to children, and stood out because of their low sugar content (12 g/100 g and 8.6 g/100 g). They also met the requirements of the FSANZ and the EU models, but not the criteria of the Keyhole model given to their low fibre content.

### Identifying nutrient profiles—results of logistic regression

In both models, RTECs marketed to children were least likely to meet the criteria of the nutrient profiles (Model 1 *OR* =0.002, *CI* =0.00–0.10; Model 2 *OR* =0.126, *CI* =0.05–0.35). Clean labelling (Model 1 *OR* =55.881; *CI* =3.89–802.13; Model 2 *OR* =4.756; *CI* =1.06–21.27) increased the likelihood of meeting the required OFCOM score, as well as the conditions of at least one nutrient profile. Organic certification was a positive determinant for a satisfactory OFCOM score (Model 1 *OR* =61.505, *CI* =4.09-923.29), whereas the existence of a whole grain claim was a positive determinant for meeting the criteria of at least one nutrient profile (Model 2 *OR* =4.282, *CI* =1.47–12.52). The results also show that the existence of a ‘health and/or nutrition claim’ did not have a significant effect on the nutrient profiles (see Table 5). The same is true for product names that referred to healthy ingredients.

**Table 5:** **Results of two logistic models of health-related information predicting nutritional value (n = 128)**

	1. Model: OFCOM score <4			2. Model:		
				At least one nutrient profile met		
	OR ^a^	(95% CI) ^b^	p ^c^	OR ^a^	(95% CI) ^b^	p ^c^
Children cereal	0.002	(0.00-0.10)	0.002	0.126	(0.05-0.35)	0.000
yes						
Healthy ingredient in product name	6.819	(0.48-97.52)	0.157	0.805	(0.27-2.42)	0.699
yes						
Whole grain claim	1.170	(0.23-5.94)	0.850	4.282	(1.47-12.52)	0.008
yes						
Health and/or nutrition claim	0.647	(0.11-3.75)	0.627	2.092	(0.73-5.99)	0.169
yes						
Clean labelling	55.881	(3.89-802.13)	0.003	4.756	(1.06-21.27)	0.041
yes						
Organic	61.505	(4.09-923.29)	0.003	4.270	(0.89-20.45)	0.069
yes						
_cons	0.044	(0.00-0.42)	0.007	0.407	(0.15-1.14)	0.087

^a)^OR = Odds Ratio ^b)^CI = Confidence Interval ^c)^Probability.

## Discussion

As expected, most highly processed German RTECs were of low nutritional quality. Only a minority qualified against any of the nutrient profiling schemes. The rate was even lower for RTECs marketed to children because of their high sugar and low fibre content. Our findings are consistent with previous studies [25, 26], such as Harris et al., who reported that none of the children’s cereals analysed in their US study qualified as nutritious products or should be marketed to children [12]. In Germany, Hampshire et al. evaluated children’s cereals against nutrition standards for in-between meals sold in schools, outlined by the Alliance for a Healthier Generation, and concluded that “the nutrient profile of the predominant part of the breakfast cereals targeted at children is not adapted to the nutritional requirements of children” [27].

The poor performance of RTECs marketed for children is relevant from a public health perspective compared with findings from the German Health Interview and Examination survey. This survey showed that children’s consumption of sugar exceeds the recommended amount by more than 200%. Over 50% of children fail to reach the recommended intake of dietary fibre [28]. Furthermore, children’s RTECs belong to the product categories that are most strongly advertised [29]. The US Federal Trade Commission reported that “in 2009, the cereals most heavily marketed to children were least nutritious” [30] *.* In addition, a substantial proportion of parents (40%) mistakenly believe that food marketed to children is nutritionally optimised for the needs of children, according to a representative survey of the Federation of German Consumer Organisations [31].

Our results showed a high prevalence of health-related claims and healthy product names on German RTECs. These items were not consistently related to the overall nutritional value of the products. Regression analysis showed that a satisfactory nutrient profile according to OFCOM was indicated by determinants which originally were not provided for the evaluation of the nutritional value of products such as cereals not marketed to children, clean labelling and organically certified. The second model, based on the criteria of different nutrient profiles, indicates that whole grain claims were one of the positive criteria to meeting at least one of the selected nutrient profiles. However, whole grain claims are not yet regulated by EU-Regulation 1924/2006. Accordingly, the whole grain content of products with respective claims varied greatly within and between our samples. In Norway, where the governmental-approved nutrient profile is in place, RTECs had on average higher whole grain contents than in Germany. As a result, we could observe significantly higher contents of fibre in Norwegian products. Moreover, the Nordic Keyhole, which requires a minimum amount of 50% whole grain, was depicted on more than half of the products with whole grain claims and even on two children products, signalling a consistent relationship between health-related on-pack information and the nutritional value of RTECs.

The situation with respect to products depicting a clean labelling claim is also complex. We found that clean labelling predicted a satisfactory nutrient profiling score in both models. However, we cannot conclude that clean labelling in general is suitable for informing consumers about the nutritional value of the product. “Free-from claims” refer to information about a variety of ingredients, such as sugar, additives, gluten, and genetic-modified organisms. Notably, however, clean labelling showed a relation to the nutrient profiling scores, whereas health and nutrition claims did not. These results demonstrate the difficulty for consumers in identifying the overall nutritional value of RTECs by external cues provided on the packaging.

Existing studies have reported similarly confusing results. Schwartz et al. demonstrated that cereals with nutrition claims did not have better overall nutrition profiles [32]. Hampshire et al. showed that low-fat cereals had less fat than those with a fat claim, but did not differ in their sugar, dietary fibre and sodium content. Moreover, cereals displaying a fibre claim did not have better nutrition profiles than those without. Only cereals advertising the claim no-sugar added scored better [15]. The assumption could be made that consumers could easily detect the true nutritional value of the product by studying the nutritional facts displayed on nearly every packaging. However, recent consumer research suggests that this information is of low relevance at the point of sale. Van Herpen and van Trijp showed that nutrition tables receive little attention and do not stimulate healthy choices, whereas simplifying heuristics, such as traffic light labels, logos, and visual cues, enhance consumers’ ability to choose more healthy products, particularly when they are under time pressure [33]. Promotion of health-supporting ingredients on the product packaging may therefore influence the health perception of the whole product, the so-called halo effect [2]. In this respect, we agree with Hughes et al. that “the promotion of unhealthy foods using claims is potentially misleading for consumers and hinders their ability to select healthier foods” [11]; p. 2154].

Adding to consumer confusion, serving sizes differed and did not allow for easy comparison of the nutritional value. Furthermore, the average serving size of children RTECs of the German 2010 sample was 30.2 grams, whereas Harris et al. reported that children consumed twice that amount of high-sugar cereals, namely 61 grams [26]. Though the latter results refer to the US it provides an indication that the serving sizes displayed on the products of our sample are likely too small. Moreover, GDA information on RTECs marketed to children was not adjusted for their needs. The discussion of whether products that are specifically designed for children should apply GDA values derived for children still remains.

Finally, we observed that the products with higher nutrient profiling scores, in most cases, were more expensive. Some affordable RTECs, which met the requirements of government-approved nutrient profiling models, were already on the market, but they were not distinguishable from other products. On the other hand, those five RTECs that met the requirements of the strictest nutrient profiling scheme, the Keyhole, belonged to the higher price category. The higher price for healthier breakfast cereals certainly is an obstacle for price-sensitive customer groups to buy those products.

Cereals from the 2012 Norwegian sample tended to have less sugar, more fibre and a better OFCOM score than German cereals. We can only speculate whether these results were caused by general differences in the country-specific food culture, or by the existence of a government-approved nutrient profile in Norway. As healthier product formulation is one of the intended positive effects from nutrient profiles [9], it is likely that both aspects are interrelated. Scientific literature indicates that a supportive food environment is an important prerequisite for the development of taste preferences and consequently healthy eating behaviour [34]. Our assumption is supported by Harris et al., who reported that children will consume low-sugar cereals when offered [26]. The better performance of the Norwegian sample demonstrates that it is possible to market RTECs with higher nutrient profiling scores.

### Limitations

The conclusions from this study should be considered within the following limitations: Formulations and packaging of the RTECs examined in this study may have changed since June 2010 and 2012. On average, however, there were no significant differences between the nutrient content of the German sample from 2010 and the German sample of 2012. We therefore assume that the obtained results are still relevant for the German market.

Our study did not include the analysis of consumer behaviour. This could be part of further investigations. In this context, several questions could be explored, such as i) country-specific differences in cereal consumption between Norwegian and German consumers; ii) consumers’ perceptions of the complex network of health-related information on products; iii) preparation mode and serving sizes of RTECs in everyday consumption; and iv) consumer perceptions of this product category and whether it is used as a staple food.

## Conclusions

The present study was the first to investigate what type of health-related information is displayed on RTECs from the German market and how this information is related to the healthiness of the products as assessed by five government-approved nutrient profiling schemes. Furthermore, the study compared the overall nutritional value of RTECs in Germany and Norway and thus in two countries with different regulations concerning nutrient profiles. Our results suggest that the implementation of a mandatory nutrient profiling scheme for products with health-related on-pack information as intended by the European regulation No 1924/2006 could contribute to a consistent relationship between health-related information on RTECs and the overall nutritional value of the product. A government-approved nutrient profile might also improve food formulation. In addition, this profile could be applied to regulate the marketing of food to children. The analysis of the Norwegian sample showed that it is possible to improve the nutritional value of RTECs. Moreover, it indicates that nutrient profiles for breakfast cereals should not only limit the sugar content, but also set minimum requirements for the fibre content. This area is lacking in the EU model.

The following improvements would enhance the quality of health-related on-pack information on highly processed RTECs.

- ● A simple nutrition labelling scheme on the front of the packaging would enable customers to identify the healthier choice.
- ● Standardised serving sizes would enable consumers to use the GDA-signalling system for comparing nutritional values between different RTECs.
- ● Product names should be included in the regulation of health-related on-pack information to prevent the use of healthy ingredients in names of products with a low nutritional value.
- ● A minimum amount of whole grain as required by the Keyhole or the IWG model should be defined if whole grain is promoted on the packaging.

Our study indicates that considerable improvement is needed to set consistent health-related information and nutritional value on product packaging in the cereal aisle.
